# Fecal Microbiota Transplantation: A Promising Therapeutic Strategy Targeting the Microbiota–Gut–Brain Axis in Anorexia Nervosa

**DOI:** 10.1002/brb3.71852

**Published:** 2026-10-28

**Authors:** Shawn L. Kwok, Yu‐Chen Xiao, Xian‐Bin Cai

**Affiliations:** ^1^ Department of Gastroenterology Shantou University Medical College Shantou Guangdong Province China; ^2^ Department of Gastroenterology The First Affiliated Hospital of Shantou University Medical College Shantou Guangdong People's Republic of China

**Keywords:** anorexia nervosa, fecal microbiota transplantation, gastrointestinal microbiome, metabolites, microbiota–gut–brain axis

## Abstract

**Purpose:**

Anorexia nervosa (AN) is a complex psychiatric disorder characterized by self‐starvation, body‐image disturbance, and serious medical complications. Because current treatments have variable outcomes and relapse is common, the microbiota–gut–brain axis has been investigated as a possible contributor to disease persistence. This review evaluates fecal microbiota transplantation (FMT) as an experimental microbiota‐directed strategy for AN.

**Methods:**

PubMed, Embase, Scopus, and Google Scholar were searched for relevant studies published from January 1990 to October 2025. Eligible evidence included human reports, animal studies, and mechanistic studies examining FMT or gut–microbiota alterations in AN and related pathways.

**Findings:**

Individuals with AN have been reported to show altered microbial diversity and changes in taxa associated with short‐chain fatty acid production and inflammation. These findings are heterogeneous and may reflect starvation, diet, medication exposure, or illness‐related physiology. Human evidence for FMT in AN is limited to small, uncontrolled studies and case reports, while most mechanistic support comes from animal models or indirect evidence from other conditions.

**Conclusion:**

Evidence for FMT in AN remains preliminary and is largely based on uncontrolled human observations and preclinical studies. FMT may alter gut microbial composition and gastrointestinal or metabolic measures, but controlled efficacy data are absent and psychiatric benefits remain unconfirmed. Well‐designed controlled trials are needed to establish its safety and clinical efficacy.

## Graphical abstract

Gut dysbiosis in anorexia nervosa may impair microbiota–gut–brain signaling through metabolic, immune, and vagal pathways. Fecal microbiota transplantation is being investigated as a potential strategy to restore host–microbiome balance, support a healthier microbial composition, and improve gastrointestinal function, appetite, and mood. Created with Figdraw.

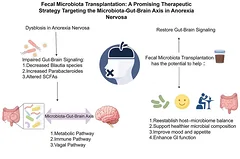

## 1 Introduction

Anorexia nervosa (AN), often referred to as anorexia, is a serious and potentially enduring psychiatric disorder. AN is characterized by restrictive food intake, an intense fear of gaining weight, a disturbance in body image (Zipfel et al. 2015). There are two subtypes of AN: restrictive subtype and binge type/purging subtype. Restrictive subtype involves extreme fasting and excessive exercise in order to lose weight. The binge type/purging subtype involves recurrent episodes of binge eating or purging behavior, such as self‐induce vomiting (Zandian et al. 2007). Despite decades of research, AN remains challenging to treat clinically, and many patients experience relapse, which can lead to serious medical complications and increase mortality. Current treatments include nutritional rehabilitation, psychotherapy, and sometimes medication. However, treatment outcomes remain variable, and relapse continues to be a significant challenge. This has driven growing interest in uncovering novel biological contributors to the illness, particularly gut–microbiota‐related pathways (Nelson et al. 2023; Quadflieg et al. 2023). At this time, the therapeutic potential of FMT in AN should be considered theoretical and hypothesis‐generating rather than clinically established. According to estimates attributed to the World Health Organization (WHO) and National Institute of Mental Health (NIMH), anorexia affects approximately 0.9% of women and 0.3% of men over their lifetimes, with approximately 8 new cases per 100,000 people annually worldwide. It is most common among adolescents and young adult women but can affect individuals of all ages and genders (GBD 2019 Mental Disorders Collaborators 2022).

In recent years, increasing attention has turned to the gut microbiota, the dynamic community of microorganisms living within the gastrointestinal tract, as a potential contributor to both the development and maintenance of AN. Emerging evidence suggests that disturbances in the gut microbiota may influence not only digestion and metabolism, but also mood regulation, behavior, and appetite, all of which are often disrupted in AN. The microbiota–gut–brain axis is a bidirectional communication network linking the central nervous system and the gastrointestinal tract, involving interactions with gut microbiota through neural, endocrine, and immune pathways. It provides a framework for examining this connection (Cryan et al. 2019; Galmiche et al. 2022). Studies have consistently reported that individuals with AN exhibit a less diverse gut microbiome. A reduction in beneficial short‐chain fatty acid‐producing bacteria may promote the overgrowth of pro‐inflammatory species, thereby contributing to intestinal inflammation and microbial dysbiosis. These alterations may be associated with key features of the disorder, including heightened anxiety, gastrointestinal discomfort, disrupted hunger signals, and difficulties with weight restoration.

Fecal microbiota transplantation (FMT) is a procedure that involves transferring processed stool from a screen healthy donor into a recipient's gastrointestinal tract to modify the recipient's gut microbiota. Clinical research has demonstrated its effectiveness in treating recurrent *Clostridium difficile* infections and Inflammatory Bowel Disease (IBD). Researchers are increasingly investigating FMT as a potential therapy for various illnesses linked to alterations in the gut flora (Wilson et al. 2021). These include metabolic diseases such as obesity and diabetes, autoimmune conditions, and certain mental health disorders which microbiome imbalances may play a role (Kwok et al. 2018; Syal et al. 2018).

Given the potential link between gut dysbiosis and behavioral symptoms in AN, FMT has been proposed as a potential adjunctive therapeutic strategy; however, current evidence remains preliminary, and its clinically efficacy has not been established. The purpose of this review is to investigate how FMT can be used as a potential treatment for anorexia nervosa by analyzing research data and the results of clinical trials. Understanding the mechanisms of AN and how FMT changes relationship within the microbiota–gut–brain axis relationship may reduce the severity of AN, particularly in patients who are not responding to traditional treatments. By drawing from both preclinical and clinical studies, we seek to clarify whether targeting the gut microbiome may represent a viable path forward in addressing this complex and often treatment‐resistant illness.

### 1.1 Literature Search and Study Selection

This review used a structured literature search and a narrative synthesis of the available evidence. The study identification and selection process was reported using the PRISMA 2020 framework. The literature search was completed in October 2025 and covered studies published between January 1990 to October 2025. Four databases were searched: PubMed, Embase, Scopus, and Google Scholar. The core concept blocks were (“fecal microbiota transplantation” OR FMT) AND (“anorexia nervosa” OR “eating disorder”). In PubMed and Embase, the searches were conducted using title/abstract field; in Google Scholar, a general keyword search was performed; and in Scopus article titles, abstracts, and keywords were searched. Reference lists of eligible articles were also examined for additional potentially relevant studies.

Inclusion criteria: Eligible reports were peer‐reviewed human, animal, or mechanistic studies that examined FMT or microbiota transfer in relation to anorexia nervosa, or that provided AN‐relevant metabolic, immunologic, neuroendocrine, or behavioral outcomes.

Exclusion criteria: Conference abstracts without full text, letters without original data, narrative opinion articles, editorials, commentaries, protocols without eligible outcome data, and studies not directly relevant to AN‐specific FMT or microbiota‐transfer mechanisms were excluded.

The searches identified 101 records: 18 from PubMed, 17 from Embase, 20 from Google Scholar, and 46 from Scopus. Records were deduplicated using PMID, DOI, and normalized title. After 35 duplicate records were removed, 66 unique records proceeded to screening. Title and abstract screening excluded 43 records. All 23 reports sought for retrieval were obtained and assessed for eligibility. Seven reports were excluded at full‐text assessment: five were reviews, editorials, or protocols without eligible primary data, and two lacked an eligible AN‐specific FMT or mechanistic outcome. Sixteen studies were included in the qualitative synthesis, comprising 3 human studies, and 13 animal or mechanistic studies (Figure 1).

**FIGURE 1:**
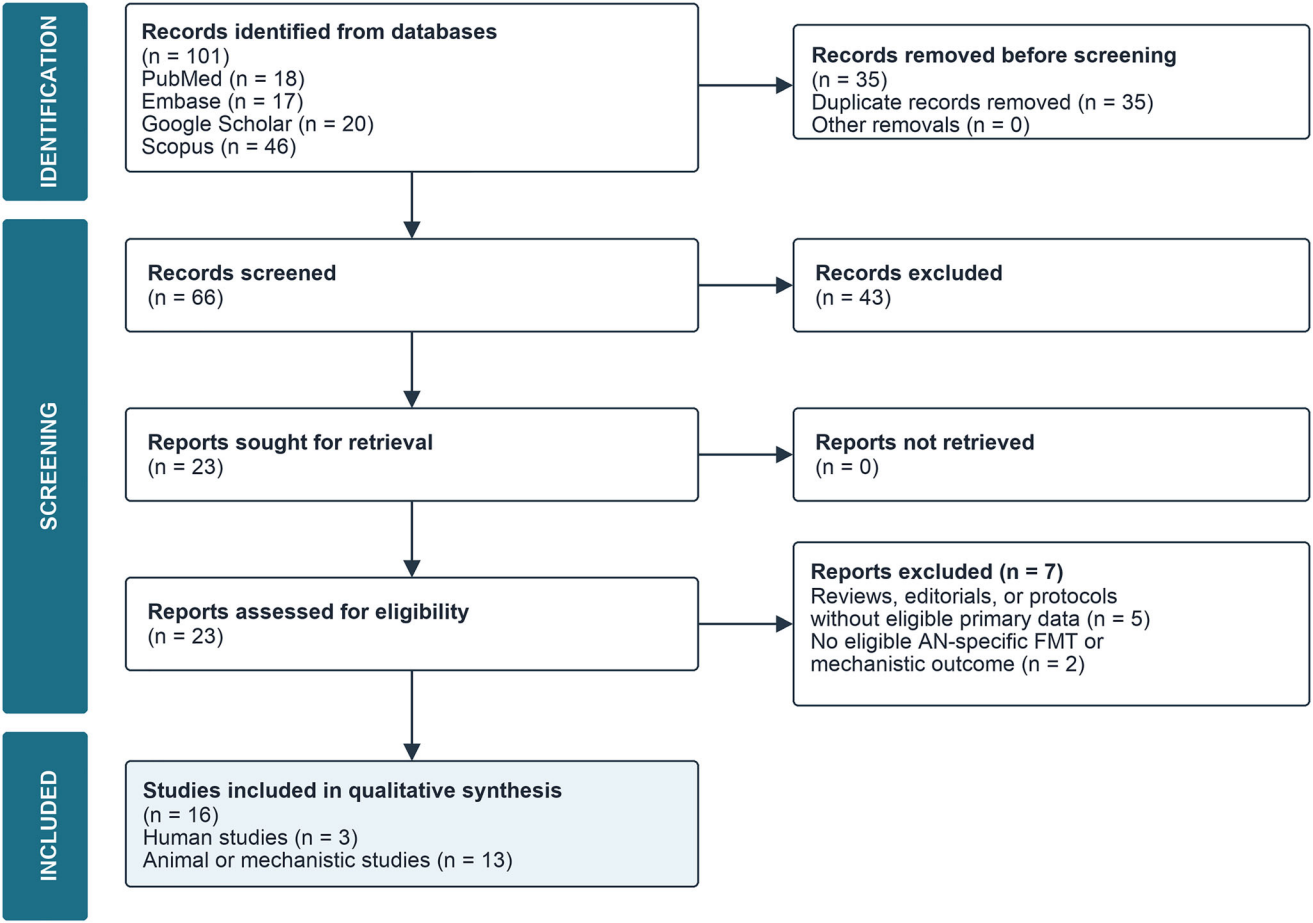
PRISMA flow diagram of the study selection process. Studies of fecal microbiota transplantation and microbiota‐related mechanisms in anorexia nervosa. Search period: first January 1990 to 31 October 2025.

No formal risk‐of‐bias assessment was conducted. This limitation should be considered when interpreting the findings, particularly given the small number of human studies and the heterogeneity of the available evidence.

### 1.2 Current Test and Medical Treatment for Anorexia Nervosa

The diagnosis of anorexia nervosa (AN) is based on clinical evaluation and DSM‐5 criteria, which include significantly low body weight, intense fear of weight gain, and distorted body image (Dang et al. 2025; Legenbauer et al. 2020). AN has two main subtypes. The first one, the restrictive type, does not involve binge eating and purging behaviors during the last three months, with patients mainly losing weight through food restriction and excessive exercise. The other subtype is the binge‐eating/purging type, in which such behaviors occur. The severity of AN is categorized as mild (BMI > 17 kg/m^2^), moderate (BMI 16‐16.99 kg/m^2^), severe (BMI 15‐15.99 kg/m^2^), or extreme (BMI < 15 kg/m^2^) (Conceição and Hilbert 2023; Dang et al. 2025). In clinical settings, patients typically undergo a comprehensive medical assessment. Laboratory tests such as a complete blood count (CBC), a metabolic panel, liver and thyroid function tests, and hormone profiles are performed to identify systemic complications related to malnutrition (Cleary et al. 2010). Electrocardiography (EKG) is used to detect cardiac arrhythmias, especially bradycardia, which is common in AN. A dual‐energy X‐ray absorptiometry (DEXA) scan is used to measure the bone mineral density (BMD), which may be assessed in patients with prolonged amenorrhea or signs of osteopenia and osteoporosis (Ahmad et al. 2023; Harris et al. 2024; Hoffman and Hall 1989).

Current treatment for AN requires a multidisciplinary approach that integrates nutritional rehabilitation, psychological support, and medical monitoring (GBD 2019 Mental Disorders Collaborators 2010). Nutritional intervention aims to restore body weight and correct metabolic imbalances and is often provided under the guidance of dietitians. Psychotherapy, particularly cognitive behavioral therapy and family‐based therapy is the mainstay of treatment, especially for adolescents and young adults. However, treatment outcomes remain suboptimal, with high relapse rates and persistent symptoms in many individuals (Carter et al. 2011; Mcintosh et al. 2005). Pharmacologic options are limited. Although selective serotonin reuptake inhibitors (SSRIs) are commonly prescribed for comorbid depression or anxiety, their efficacy in underweight individuals with AN is unclear (Marvanova and Gramith 2018). Atypical antipsychotics such as olanzapine have been investigated for their potential to promote weight gain and reduce obsessive thinking, but results are inconsistent, and no medications are currently approved by the FDA specifically for AN (Crow et al. 2009). Due to the limited effectiveness of conventional therapies, novel strategies such as fecal microbiota transplantation (FMT) have emerged as potential adjunctive treatments. These approaches reflect a growing interest in targeting gut dysbiosis and the microbiota–gut–brain axis in AN pathophysiology.

### 1.3 Dysbiosis in Anorexia Nervosa: Mechanisms and Clinical Implications

Recent studies have reported significant alterations in the gut microbiota of patients with AN, a condition often described as dysbiosis. Nutritional deprivation significantly alters gut microbiota composition, but these microbial changes may also actively perpetuate the illness (De Clercq et al. 2019). Although several studies report reduced *Faecalibacterium* and increased *Parabacteroides* in AN, the overall microbial patterns across published datasets remain heterogeneous and directional changes are not uniform. *Faecalibacterium* plays an important role in maintaining gut barrier integrity, regulating immune responses, and breaking down complex carbohydrates. A reduction in its abundance may compromise mucosal health, weaken anti‐inflammatory signaling, and disrupt neuroendocrine communication (Ramirez‐Farias et al. 2009; Turroni et al. 2014). Variability in findings may reflect methodological differences, such as sequencing platforms, stool versus mucosal sampling sites, and statistical approaches. Differences in patient characteristics, including nutritional status, illness duration, and comorbidities, as well as regional dietary and geographic factors, may also contribute to this variability. Therefore, functional metrics such as SCFA biosynthetic capacity, mucosal barrier integrity, bile acid conversion pathways, and neuroactive metabolite potential may be more clinically meaningful than focusing on specific taxa alone.

It is also important to note that microbial patterns observed in AN may reflect the physiological consequences of starvation, including severely reduced caloric intake, low fiber and carbohydrate availability, slowed intestinal transit, and metabolic energy conservation rather than a disease‐specific pathogenic signature (Sonnenburg and Bäckhed 2016). Therefore, at least a portion of the observed “dysbiosis” may represent secondary adaptive responses to extreme nutritional deprivation rather than a primary driver of AN pathophysiology. This distinction has important implications when considering broad microbial interventions like FMT. It also remains unclear whether dysbiosis is a driver of AN pathology, a consequence of starvation and restrictive eating or a bidirectional feedback effect.

Notably, *Faecalibacterium prausnitzii* and *Akkermansia muciniphila* play distinct roles in maintaining intestinal health. *F. prausnitzii* produces butyrate, a SCFAs that provides energy to intestinal cells, reduces inflammation, and supports gut barrier integrity. *A. muciniphila* primarily utilizes mucin and contributes to mucus layer regulation. Alterations in these bacteria may affect metabolic and immune functions, although their roles in AN remain incompletely understood (Bottacini et al. 2014).

Clinical research has shown that individuals with anorexia nervosa (AN) experience significant alterations in their gut microbiota. These changes include a reduction in beneficial bacteria such as *Bifidobacterium*, *Lactobacillus*, *Firmicutes*, *Ruminococcaceae*, *Roseburia*, *Clostridium*, *Anaerostipes, Faecalibacterium*, and *Peptostreptococcaceae*, alongside an increase in harmful microbes such as *Proteobacteria, Lachnospiraceae, Bacteroidaceae, Coriobacteriaceae, Enterobacteriaceae, Methanobrevibacter smithii, Actinobacteria, Verrucomicrobia*, and *Methanobrevibacter* (Hiippala et al. 2018). This imbalance disrupts the production of short‐chain fatty acids (SCFAs), including butyrate, which plays a crucial role in maintaining the gut barrier and regulating inflammation. Lower SCFA levels can increase intestinal permeability, allowing inflammation to spread systemically (Parada Venegas et al. 2019; Zhuang et al. 2019). Additionally, gut microbiota influence the production of neurotransmitter such as serotonin and dopamine, which regulate mood and appetite. A decline in beneficial bacteria that support serotonin synthesis may worsen anxiety and depression in AN. Psychological stress further reinforces restrictive eating behaviors, creating a vicious cycle (Hainer et al. 2006).

Conversely, certain microbial taxa, such as *Akkermansia muciniphila*, *Clostridium coccoides*, *Clostridium ramosum*, and various *Enterobacteriaceae* species, are elevated in AN and may exacerbate dysfunction. *Akkermansia muciniphila*, for example, degrades mucin, a component of the protective intestinal mucus layer, potentially thinning this layer and increasing epithelial permeability (Dupont et al. 2020; Meng et al. 2020; Wang et al. 2022). This could allow harmful microbial products such as lipopolysaccharides (LPS) to enter the circulation, triggering inflammation and affecting brain function via neuroimmune pathways. These microbial shifts also disrupt the microbiota–gut–brain axis, impairing the production of SCFA including butyrate, propionate, and acetate, which influence satiety, immunity, and neurotransmitter synthesis. Reduced SCFA levels may worsen gastrointestinal symptoms, anxiety, and appetite dysregulation. Animal studies further support this link, as transferring AN‐associated microbiota into germ‐free mice leads to reduced weight gain and behavioral changes, suggesting that gut microbes directly contribute to the metabolic and neuropsychiatric aspects of the disorder (Liu et al. 2021; Martin‐Gallausiaux et al. 2021; Quagebeur et al. 2023). Together, these findings indicate that gut dysbiosis in AN is not just a consequence of restricted eating but may actively sustain the disorder through metabolic, immune, and brain‐related mechanisms. Understanding this connection could inform treatments such as probiotics, prebiotics, and fecal microbiota transplantation (FMT) to restore microbial balance and alleviate symptoms.

### 1.4 Hypothalamic–Pituitary–Adrenal (HPA) Axis in Anorexia Nervosa

The hypothalamic–pituitary–adrenal (HPA) axis is a central stress‐response system, and it has been found to be significantly dysregulated in patients with AN. The HPA contributes to the persistence of neuroendocrine, metabolic, and behavioral disturbances. In individuals with AN, prolonged caloric restriction, combined with psychological stress, results in sustained activation of the HPA axis, leading to elevated levels of corticotropin‐releasing hormone (CRH), adrenocorticotropic hormone (ACTH), and cortisol (Chan and Mantzoros 2005; Licinio et al. 1996). This hypercortisolemic state reflects not only an adaptive mechanism to stress but also a maladaptive cycle that perpetuates catabolism, hinders weight gain, and exacerbates mood disturbances. Elevated cortisol promotes gluconeogenesis, muscle breakdown, and lipolysis while simultaneously inhibiting bone formation, reproductive function, and immune competence (Amasi‐Hartoonian et al. 2022).

From a physiological standpoint, the hypothalamus secretes CRH in response to stress, which in turn stimulates the anterior pituitary to release ACTH, thereby driving cortisol production by the adrenal cortex (Itoi et al. 2004). In AN, several studies have shown increased basal cortisol and altered diurnal secretion patterns, suggesting a breakdown in the normal negative feedback regulation of the axis. Moreover, CRH itself exerts anorexigenic effects by suppressing appetite and enhancing anxiety‐like behavior, which may reinforce the restrictive eating patterns seen in AN (Farzi et al. 2019; Licinio et al. 1996). This creates a vicious cycle in which chronic starvation amplifies stress signaling and elevated stress hormones further sustain disordered eating behaviors.

Additionally, recent research has suggested that HPA axis dysregulation in AN may interact bidirectionally with the gut microbiota, forming a neuroendocrine‐microbiota feedback loop. Cortisol can influence gut permeability, mucosal immunity, and microbial composition, while microbial metabolites such as short‐chain fatty acids (SCFAs) and certain neurotransmitter‐like compounds may then affect HPA axis activity via the vagus nerve and immune modulation (Kobayashi et al. 2022; Misiak et al. 2020; Van De Wouw et al. 2017). Hence, alterations in gut flora commonly seen in AN may not only be a consequence of nutritional deprivation but also may also contributed to HPA axis dysregulation and psychological symptomatology.

Even after partial or full weight restoration, abnormalities in cortisol secretion and stress reactivity may persist, suggesting that HPA axis dysfunction could represent a trait vulnerability in some individuals with AN. These findings point toward the need for more integrated treatment strategies that target both nutritional rehabilitation and the neuroendocrine stress axis. Such strategies may include pharmacological agents that modulate CRH signaling, psychotherapeutic approaches to reduce stress, and microbiota‐based therapies aimed at restoring gut–brain homeostasis (Wei et al. 2021).

### 1.5 Microbiota–Gut–Brain Axis in Anorexia Nervosa

Emerging research has revealed the profound role of gut health in regulating brain function, particularly through the influence of the gut microbiota on the synthesis and modulation of neurotransmitters and neuropeptides (Cryan and Dinan 2012; Foster et al. 2017). The gut microbiota comprises trillions of microorganisms and interacts with the central nervous system through the microbiota–gut–brain axis, which is a complex and dynamic bidirectional communication network. Disruption of this system, often due to microbial imbalance or dysbiosis, has been implicated in various neuropsychiatric conditions, including anorexia nervosa (AN) (Breton et al. 2019; Mörkl et al. 2020; Prochazkova et al. 2021).

### 1.6 Metabolic Pathway

One major pathway through which the gut communicates with the brain involves the production and regulation of neurotransmitters and neuropeptides, which are crucial for neural development, emotional regulation, and behavioral control (Figure 2) (Mayer et al. 2014). Imbalances in key neurotransmitters such as serotonin, dopamine, and norepinephrine, as well as CRH and signaling molecules including neuropeptide Y (NPY), leptin, ghrelin, endorphins, melanocortin, and cholecystokinin, have been consistently observed in patients with AN (Barbarich et al. 2003; Kaye et al. 2009). These neurochemical disruptions can significantly affect mood regulation, appetite control, and impulse inhibition, all of which are critical in the pathology of restrictive eating behaviors characteristic of AN (Kaye et al. 2005).

**FIGURE 2:**
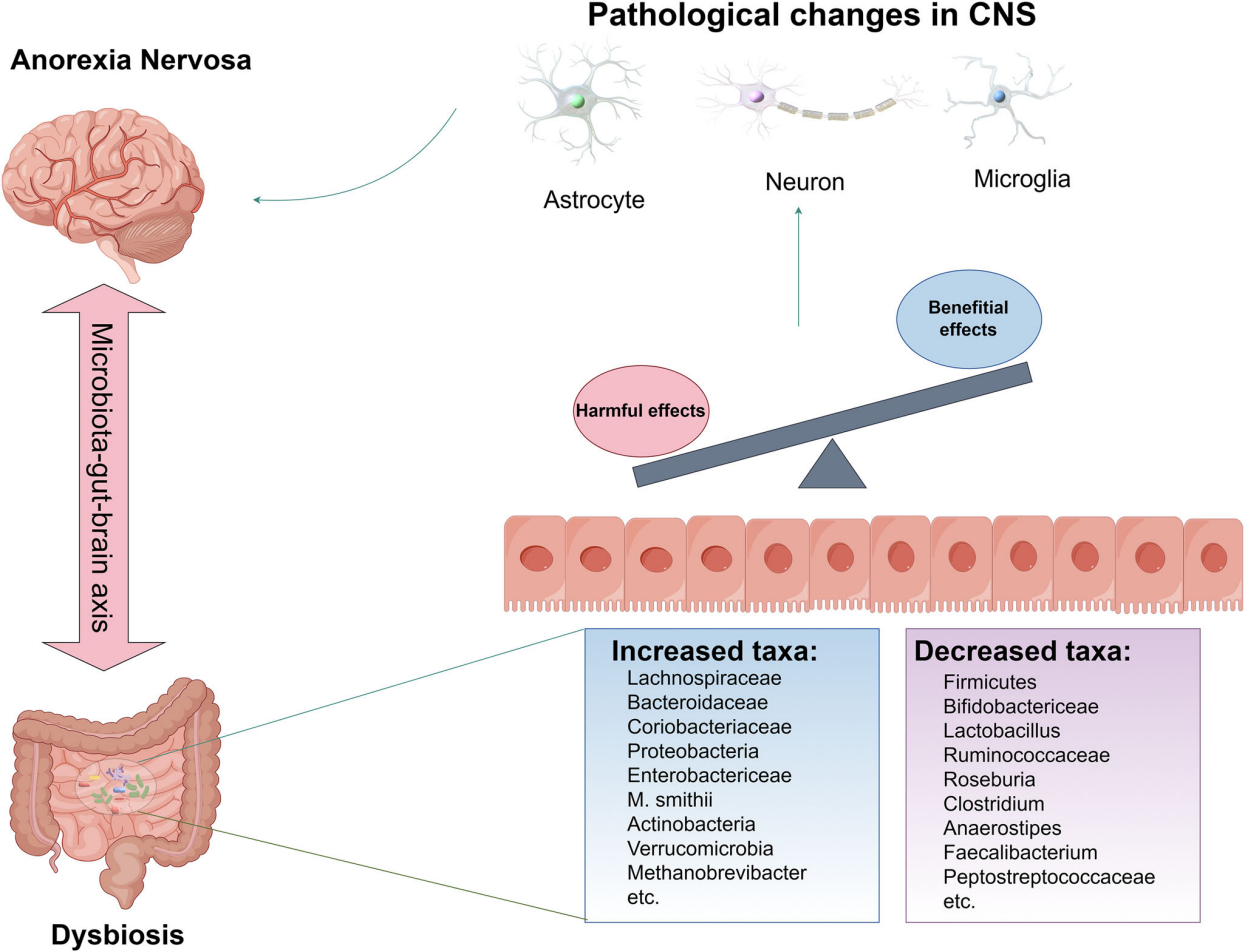
The association between dysbiosis and central pathological changes during the development of anorexia nervosa. CNS, central nervous system. Created with Figdraw.

Moreover, these neurochemical imbalances exert their effects on several brain regions that are central to eating behavior and self‐regulation. The hypothalamus, which plays a central role in integrating hormonal signals related to hunger and satiety, particularly those of leptin and ghrelin, can become dysregulated in AN (Gautron and Elmquist 2011). As a result, the brain may misinterpret or fail to respond to physiological hunger cues, reinforcing food avoidance (Kaye et al. 2009).

Similarly, the prefrontal cortex, which is responsible for executive function and self‐control, may also show impaired activity and undermining the ability to make appropriate dietary decisions (Zastrow et al. 2009). The insular cortex is involved in interoception and taste processing, and it may also become altered in its ability to accurately assess internal bodily states and sensory inputs, including hunger, satiety, and taste perception (Kerr et al. 2016). These combined effects result in distorted perceptions of eating that fuel restrictive behavior and perpetuate the illness (Frank et al. 2012). Notably, gut dysbiosis may be a key upstream factor driving many of these neurochemical disturbances. Certain microbial species are capable of synthesizing or modulating neuroactive compound, including short‐chain fatty acids, tryptophan metabolites, and γ‐aminobutyric acid (GABA), which can influence brain function via the vagus nerve, immune signaling, and the circulatory system (Clarke et al. 2014; Cryan and Dinan 2012; Gérard 2016). When beneficial microbes are depleted or pathogenic species overgrow, the delicate neurochemical balance in the brain can be disturbed, potentially initiating or exacerbating the psychological and behavioral symptoms of AN (Mörkl et al. 2020).

These findings underscore the central role of the microbiota–gut–brain axis in the development and maintenance of anorexia nervosa. By influencing neurotransmission, neuroendocrine signaling, and central nervous system function, the gut microbiota emerges not only as a mediator but also as a potential therapeutic target for intervention (Dinan and Cryan 2017). While mechanistic insights from obesity, depression, and IBD models provide valuable hypothesis‐generating frameworks, these data represent indirect extrapolations rather than proven causal mechanisms in AN. Host metabolic state, nutritional status, immune tone, and endocrine signaling differ profoundly between starvation phenotypes and metabolic excess states. For example, taxa such as Akkermansia may play divergent physiological roles in an underweight, catabolic AN patient compared with an obese host. Therefore, cross‐condition microbiome findings should be interpreted cautiously and viewed primarily as conceptual scaffolding rather than direct evidence of FMT efficacy in AN.

### 1.7 Immune Pathway

Immune dysregulation plays a significant role in the microbiota–gut–brain axis, particularly in anorexia nervosa (AN). Several environmental and physiological factors, such as chronic stress, an inadequate diet, micronutrient deficiencies, antibiotic use, and comorbid conditions including autoimmune diseases (e.g., inflammatory bowel disease, celiac disease), metabolic disorders (e.g., type 1 diabetes, rheumatoid arthritis), and liver dysfunction, can contribute to gut microbiota dysbiosis and increased intestinal permeability, a condition often referred to as “leaky gut syndrome” (Kinashi and Hase 2021; Stewart et al. 2017). This compromised gut barrier may allow luminal antigens, endotoxins (e.g., lipopolysaccharides) and pro‐inflammatory cytokines such as interleukin‐6 (IL‐6) and tumor necrosis factor‐alpha (TNF‐α) to translocate into the systemic circulation and potentially cross the blood–brain barrier (Figure 3) (Braniste et al. 2014; Kelly et al. 2015; Mass et al. 2008).

**FIGURE 3:**
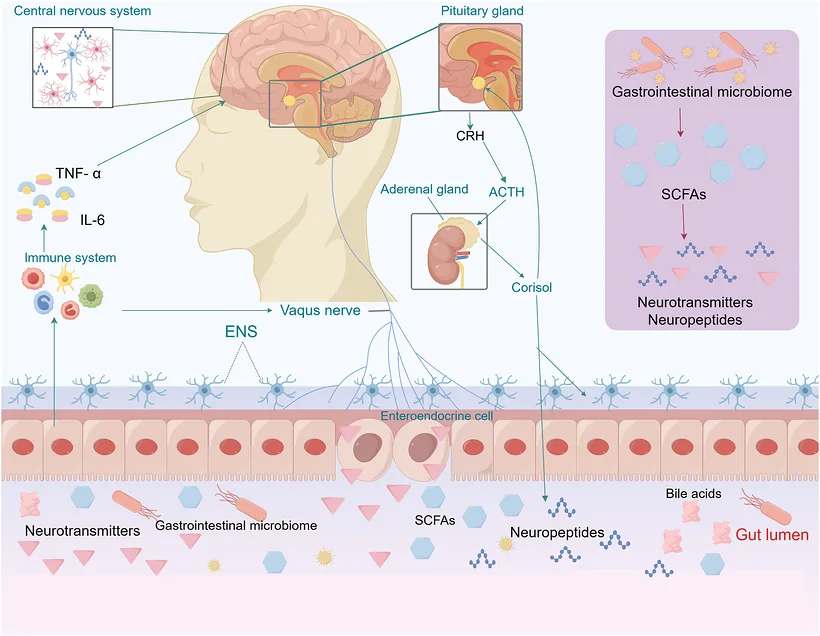
The intricate pathways of gut‐to‐brain communication in anorexia nervosa. HPA, hypothalamic–pituitary–adrenal; SCFAs, short‐chain fatty acids; IL‐6, interleukin‐6; TNF‐α, tumor necrosis factor‐alpha; ENS, enteric nervous system. Created with Figdraw.

In the central nervous system (CNS), these inflammatory mediators may trigger neuroinflammation, disrupt neuronal signaling, and interfere with the synthesis and regulation of key neurotransmitters and neuropeptides (Dantzer et al. 2008; Erny et al. 2015). This kind of dysregulation has been linked to mood disturbances, heightened anxiety, cognitive impairment, and obsessive–compulsive behaviors (OCD), which are core psychological features frequently observed in AN (Kaye et al. 2009). Moreover, altered brain function in AN patients can contribute to appetite suppression and an aversion to food intake, exacerbating nutritional deficiencies and perpetuating gut dysbiosis. This creates a self‐reinforcing cycle in which microbiota imbalance, immune activation, and neuropsychiatric symptoms reinforce one another (Frank et al. 2012). Therefore, restoration of a healthy gut microbiome and attenuation of low‐grade systemic inflammation may offer a promising therapeutic avenue in the treatment of anorexia nervosa (Dinan and Cryan 2017; Prochazkova et al. 2021).

### 1.8 Vagal Pathway

The vagus nerve plays a critical role in the microbiota–gut–brain (MGB) axis, acting as a bidirectional communication conduit between the gut and the central nervous system (Bonaz et al. 2018). Approximately 80%–90% of vagal fibers are afferent and transmit sensory information from the gastrointestinal tract to brain regions such as the nucleus tractus solitarius (NTS), hypothalamus, and insula, which are involved in regulating appetite, emotion, and stress responses. This neural feedback loop is essential for interpreting hunger and satiety signals, modulating mood, and maintaining homeostasis. In AN, dysbiosis can impair vagal signaling, and this impairment contributes to aberrant interpretations of internal bodily signals, such as hunger, fullness, and nausea, which are frequently reported in AN patient (Monteleone et al. 2008).

Clinical studies indicate that certain microbial metabolites, such as short‐chain fatty acids (SCFAs) including butyrate and propionate, normally stimulate vagal afferents to promote feelings of satiety and emotional well‐being (Dalile et al. 2019). However, in dysbiosis, decrease SCFA production and increased gut inflammation (e.g., elevated lipopolysaccharide, IL‐6, and TNF‐α) may blunt this signaling or activate vagal fibers inappropriately, leading to anxiety, mood disturbances, and food avoidance behaviors (Figure 3) (Kelly et al. 2015).

In addition, the gut microbiota also influences the production of key neurotransmitters such as serotonin, dopamine, and GABA, which are synthesized in the gut and can act via the vagus nerve (Strandwitz 2018). For example, serotonin, over 90% of which is produced in the gut, can stimulate 5‐HT3 receptors on vagal afferents and thereby affect brain circuits involved in mood and cognition (O'mahony et al. 2015). Dysbiosis may lead to reduced neurotransmitter availability or altered receptor expression, contributing to heightened anxiety, depression, and obsessive–compulsive behaviors, which are commonly observed in AN (Kaye et al. 2009).

Experimental studies provide further support: vagotomy in mice has been shown to block behavioral changes induced by gut microbial transplantation, indicating that the vagus is a necessary pathway for gut–brain effects (Bercik et al. 2011). Similarly, functional MRI studies in patients with AN have revealed altered activation in vagus‐associated brain regions during interoceptive tasks.

Dysbiosis also promotes increased intestinal permeability, enabling the translocation of endotoxins and immune activation. These pro‐inflammatory signals can affect vagal tone and interfere with its regulation of the hypothalamic–pituitary–adrenal (HPA) axis, leading to elevated cortisol, stress sensitivity, and disruption of appetite control (Foster et al. 2017). This results in a self‐perpetuating cycle: malnutrition and food restriction in AN reduce vagal tone and microbial diversity, worsening dysbiosis, further impairing vagal signaling, and deepening neuropsychiatric symptoms. In summary, vagal nerve dysfunction may be triggered or exacerbated by gut dysbiosis and appears to play a significant role in the emotional, cognitive, and feeding disturbances observed in AN. Restoring healthy gut microbiota and enhancing vagal tone may therefore represent key therapeutic targets for interrupting this pathological cycle and improving both gastrointestinal and psychiatric outcomes in AN.

### 1.9 Gut Microbiota‐Derived Metabolites and Anorexia Nervosa

The gut microbiota plays a significant role in numerous physiological processes, including nutrient metabolism, immune modulation, and neurochemical signaling (Cryan and Dinan 2012). Increasing evidence suggests that gut dysbiosis contributes significantly to the pathophysiology of anorexia nervosa (AN) by altering the production of key microbial metabolites that influence gastrointestinal and neuropsychiatric function (Attia 2010; Mack et al. 2016). In healthy individuals, commensal bacteria help in the digestion of complex carbohydrates and the synthesis of metabolites that serve as precursors to neurotransmitters and neuropeptides.

Dysbiosis in patients with AN is often characterized by reduced microbial diversity and altered composition, which can disrupt these pathways. Specifically, clinical studies have shown a decrease in the relative abundance of *Firmicutes* and *Bacteroidetes* in individuals with AN (Borgo et al. 2017; Kleiman et al. 2015). This shift leads to impaired synthesis of important metabolites such as short‐chain fatty acids (SCFAs), bile acids, and neuroactive compounds. SCFAs include butyrate, acetate, and propionate and are among the most extensively studied microbial metabolites. These molecules perform several critical functions, such as providing energy to colonocytes, strengthening the intestinal barrier, regulating local and systemic immune responses, and influencing gene expression via histone deacetylase (HDAC) inhibition (Dalile et al. 2019). Reduced SCFA production, as observed in AN, has been associated with increased intestinal permeability, also known as “leaky gut,” which allows microbial products such as lipopolysaccharide (LPS) to translocate into the systemic circulation (Kelly et al. 2015). LPS binds to Toll‐like receptor 4 (TLR4) on immune cells, triggering the release of proinflammatory cytokines, including tumor necrosis factor‐alpha, interleukin‐1β, and interleukin‐6 (Cani et al. 2007). This systemic inflammation may further compromise gut barrier function and exacerbate both gastrointestinal and neuropsychiatric symptoms in AN. Beyond immune modulation, gut bacteria are also involved in the biosynthesis of key neurotransmitters and neuromodulators, including dopamine, gamma‐aminobutyric acid (GABA), and serotonin (5‐HT) (Strandwitz 2018).

Over 90% of the serotonin in the human body is produced in the gut (O'mahony et al. 2015). These neuroactive metabolites influence brain function via the vagus nerve, the bloodstream, and local enteroendocrine pathways. Dysbiosis‐induced disruption in the levels of these compounds is thought to contribute to mood disturbances, anxiety, and OCD, which are core features of AN (Dargenio et al. 2023). For instance, reduced serotonin levels may impair satiety signaling and emotion regulation, potentially reinforcing the restrictive eating patterns and emotional dysregulation observed in patients. Given these associations, therapeutic strategies aimed at restoring microbial balance are of growing interest. Fecal microbiota transplantation (FMT) has emerged as a promising intervention with the potential to re‐establish a diverse and functionally robust microbiota (Prochazkova et al. 2019). Preliminary evidence suggests that FMT may increase SCFA production, enhance gut barrier integrity, and normalize neuroactive metabolite profiles. Through these mechanisms, FMT may offer dual benefits in treating both the gastrointestinal abnormalities and psychiatric symptoms of AN. However, FMT remains a broad and relatively non‐specific microbial intervention, and its effects on these mechanistic pathways in AN remain unclear. The proposed mechanistic pathways potentially linking the gut microbiota to anorexia nervosa are summarized in Table 1.

**TABLE 1:** Summary of proposed mechanistic pathways potentially linking to gut microbiota to AN.

Mechanistic domain	Microbial influence mechanism	Evidence base	Relevance to AN	Evidence strength
SCFA/metabolic signaling	SCFA regulate satiety hormones, inflammation and energy utilization	Primarily animal + limited human metabolic correlates	May influence nutritional tolerance + refeeding physiology	Preliminary/exploratory
HPA axis/neuroendocrine	Microbial metabolites modulate stress hormone signaling and CRH‐cortisol feedback	Animal mechanistic + biomarker inference	Could contribute to persistent anxiety/stress reactivity in AN	Exploratory/non causal
Immune/barrier integrity	Dysbiosis may increase permeability + pro‐inflammatory signaling	Animal + human biomarker association	May contribute to chronic low‐grade neuroimmune activatioon	Limited/indirect
Vagal/neurocircuit signaling	Microbial products influence vagal afferent input and interoceptive signaling	Mechanistic animal data	Could alter hunger/satiety interpretation and GI‐visceral input processing	Hypothesis—generating only

### 1.10 The Interactions Between Gut Microbiota and Antidepressants

The relationship between selective serotonin reuptake inhibitors (SSRIs) and the intestinal microbiota is particularly important in the context of anorexia nervosa (AN), especially given the high prevalence of gastrointestinal symptoms and psychiatric comorbidities in this population. Approximately 90% of the body's serotonin (5‐HT) is synthesized primarily by enterochromaffin cells in the gut, under the influence of gut microbial metabolites (Landini et al. 2023).

SSRIs such as fluoxetine, sertraline, escitalopram, paroxetine, and citalopram are commonly prescribed to patients with AN to alleviate symptoms of anxiety, depression, and obsessive–compulsive behavior (Marvanova and Gramith 2018). They block serotonin reuptake at synaptic terminals in the central nervous system, thereby enhancing serotonergic signaling. However, the clinical efficacy of these medications in underweight or severely malnourished patients with AN remains limited. Paradoxically, SSRIs may exacerbate anxiety and food aversion in some cases (García‐Alba 2004). Despite these limitations, SSRIs may still be valuable during the weight restoration phase in patients with AN, potentially helping to reduce relapse rates and support emotional regulation (Ghenciulescu et al. 2020). Moreover, SSRIs can influence gastrointestinal function via peripheral serotonin receptors. For instance, sertraline and fluoxetine have been shown to enhance gastric motility and accelerate gastric emptying, which may provide symptomatic relief from common GI complaints in patients with AN (Tang et al. 2024).

On the other hand, the Intestinal microbiota also contributes significantly to serotonin metabolism. Certain strains of *Lactobacillus* (e.g., *L. rhamnosus*) and *Bifidobacterium* (e.g., *B. infantis*) enhance the conversion of dietary tryptophan into serotonin and other neuroactive metabolites. These intestinal microbiota play a key role in the gut–brain axis (Gao et al. 2020; Liu et al. 2020). Gut bacteria also ferment indigestible carbohydrates to produce short‐chain fatty acids (SCFAs), such as acetate, propionate, and butyrate. SCFAs stimulate tryptophan hydroxylase 1 in EC cells and promote 5‐HT synthesis (Margolis et al. 2021). However, patients with AN often exhibit reduced SCFA production due to both microbial dysbiosis and decreased food intake. This may result in lower serotonin synthesis and contribute to the emotional dysregulation, depression, and anxiety commonly seen in AN (Anton‐Păduraru et al. 2023). While 5‐HT produced in the gut does not readily cross the blood–brain barrier, it may influence the brain function indirectly through peripheral signaling pathways (Kopeikina et al. 2020).

Furthermore, reduced microbial diversity in AN may contribute to lower SCFA levels, impairs gut barrier integrity, and increased systemic inflammation, all of which may be relevant to psychiatric symptoms (Wei et al. 2022). Emerging evidence suggests that microbial‐targeted therapeutics could influence responses to SSRIs. FMT from healthy donors may help restore microbial diversity and beneficial SCFA‐producing bacteria (Liu et al. 2023). Theoretically, these microbial changes could influence serotonin‐related pathways and SSRI responsiveness, although this has not been established in patients with AN. Although findings from other psychiatric and gastrointestinal disorders provide a rationale for further investigation, they constitute indirect evidence and cannot establish the efficacy of FMT as an adjunctive treatment for AN.

### 1.11 Fecal Microbiota Transplantation in Anorexia Nervosa and Malnutrition

The included human studies of fecal microbiota transplantation in anorexia nervosa are summarized in Table 2.

**TABLE 2:** Summary of included human studies of FMT in anorexia nervosa.

Study and year	Design/*N*	Population	FMT details	Outcomes reported	Limitations
de Clercq et al. (2019)	Case report (*N* = 1)	Adult with recurrent underweight and AN history	Single course; donor FMT	13.8% weight ↑ over 36 weeks; ↑ A. muciniphila, ↑ acetate/butyrate	Uncontrolled; cannot infer causality
Xie et al. (2022)	Case report	24 y/o female with T1DM + malnutrition (AN phenotype)	Frozen FMT x 2 via NJ tube	Improved constipation, weight ↑, improved glycemic control; microbial shifts related to weight/mood taxa	AN + T1DM confounding; uncontrolled
Procházková et al. (2019)	Case report (*N* = 1)	37‐year‐old woman with severe and enduring AN and SIBO	Serial FMT from a health donor	↑ bacterial richness and diversity; ↑ SCFA levels; improved intestinal barrier function; no clinical improvement	Single‐patient case report; no control group; findings cannot establish clinical efficacy

Interest in FMT as a potential therapy for anorexia nervosa (AN) and malnutrition has grown due to increasing recognition of the gut–brain axis and its influence on metabolic and psychological health (Cryan et al. 2019). AN is frequently associated with gut dysbiosis, which may contribute to both gastrointestinal symptoms and neuropsychiatric manifestations. FMT may help correct this intestinal imbalance and thereby support recovery. de Clercq et al. (2019) reported a single case in which a patient showed a 13.8% increase in body weight over 36 weeks following FMT despite stable caloric intake. However, as an uncontrolled observation, this association does not imply causality and should be interpreted as hypothesis‐generating. The weight gain was primary due to increased body fat and was associated with changes in microbial composition. The data showed an increase in *Akkermansia muciniphila* and short‐chain fatty acids (SCFAs), including acetate and butyrate, which are critical for metabolic regulation and intestinal health (De Clercq et al. 2019).

Another clinical case study by Xie et al. (2022) reported on a 24‐year‐old female with type 1 diabetes and malnutrition who showed improvements in constipation, nutritional status, and glycemic control after two rounds of frozen FMT via the nasojejunal route. The patient's weight increased from 25 kg resulting in a healthier BMI, and she achieved better glycemic control. No adverse effects were reported. Microbiome analysis showed an increased abundance of *Blautia*, a genus positively associated with weight gain and a decreased abundance of *Parabacteroides*, which had previously been linked to weight suppression via succinate production. These microbiota shifts included taxa previously associated with mood regulation in other literature, but this does not establish that FMT directly improved mood in this individual. These observations remain preliminary and speculative (Nikolova et al. 2021; Xie et al. 2022).

Prochazkova et al. (2019) also reported a case involving a 37‐year‐old female with long‐standing AN who received FMT via a nasogastric tube. Following the treatment, the patient showed improved gut microbial diversity, enhanced intestinal barrier function, and increased levels of short‐chain fatty acids, particular butyrate. There were no significant changes in mood or eating behaviors during the follow‐up (Prochazkova et al. 2019). The case highlights the potential of FMT to restore gut health in patients with AN and supports further investigation into its therapeutic role.

Selected preclinical animal studies examining microbiota transfer in anorexia nervosa are summarized in Table 3. These preclinical findings should be interpreted as exploratory mechanistic evidence only and not as predictors of clinical efficacy in humans

**TABLE 3:** Summary of selected preclinical animal studies.

STUDY AND YEAR	Model	Microbiota source	Key findings	Limitations
Roubalová et al. (2024)	Germ‐free mice	AN vs. healthy microbiota	AN microbiota → ↓ weight gain + metabolic gene shifts	Not predictive of human outcomes
Maschek et al. (2025)	Antibiotic‐treated mice	AN vs. healthy donor	AN microbiota ↓ food intake + altered satiety hormones; partially reversed by healthy donor FMT	Exploratory; behavioral complexity not comparable
Kooij et al. (2024)	Tyrosine hydroxylase‐Cre rats	AN donor microbiota	No significant changes in anxiety or cognitive flexibility	No significant behavioral effects observed in this model

Meanwhile, a systematic review by Carbone et al. (2021) concluded that patients with AN consistently display altered gut microbiota compared to healthy controls, reinforcing the theoretical rationale for FMT interventions. Animal models provide exploratory insight into microbial influences on behavior and metabolism, but they do not establish efficacy in humans. In a landmark study by Roubalová et al. (2024), germ‐free mice colonized with microbiota from AN patients showed significantly reduced weight gain and altered gene expression patterns related to metabolism and appetite compared with mice receiving healthy donor microbiota. Similarly, a cross‐over mouse study by Maschek et al. (2025) demonstrated that transferring microbiota from AN patients into antibiotic‐treated mice reduced food intake and altered hormone profiles related to satiety. These effects were partially reversed following FMT from healthy donors (Maschek et al. 2025). Interestingly, another study by Kooij et al. (2024) in tyrosine hydroxylase‐Cre rats showed no significant changes in anxiety or cognitive flexibility following FMT from AN patients, suggesting that microbial contributions to psychological symptoms may be complex and context‐dependent.

Collectively, both preclinical and early clinical data suggest that FMT warrants further investigation as a potential adjunctive therapy for AN, although its effects on microbial balance, metabolic health, and psychological outcomes remain uncertain. However, more robust randomized controlled trials are needed to establish its long‐term safety, efficacy, and mechanisms of action.

### 1.12 Predictors of Response to Fecal Microbiota Transplantation in Anorexia Nervosa

Several factors may influence the therapeutic response to FMT in individuals with AN. These include disease severity, donor selection, stool type (fresh vs. frozen), route of administration, treatment timing and frequency, pre‐treatment with antibiotics, and the patient's baseline gut microbiota profile. Patients with more severe or long‐standing AN often exhibit distinct microbial imbalances, which may affect how well the donor microbiota engrafts and functions. A study by Hata et al. (2019) demonstrated that fecal microbiota from AN patients, when transplanted into germ‐free mice, induced reduced food intake and anxiety‐like behavior, suggesting that the AN‐associated microbiota itself may contributes to disease pathology and might influence FMT outcomes (Hata et al. 2019). Donor characteristics may also crucial in determining FMT effectiveness.

Research published by Wilson et al. (2021) in metabolic syndrome and IBD has found that pooled‐donor FMT yields better microbial diversity and engraftment than single‐donor approaches. This strategy increases the chances of delivering key beneficial microbes and it could potentially be useful in AN where the intestinal environment is often severely depleted due to malnutrition (Wilson et al. 2021). However, these findings were obtained in populations without AN, and their applicability to severely malnourished patients remains uncertain because starvation may alter the intestinal environment and microbial engraftment.

The type of stool used is also important. While frozen stool offers logistical convenience and retains efficacy, some studies suggest that fresh stool may better preserve sensitive anaerobes such as *Faecalibacterium prausnitzii*, which plays key anti‐inflammatory roles (Nishida et al. 2018).

The route of FMT delivery can further influence colonization success. Oral capsules are less invasive and generally preferred by patients, but colonoscopic or nasoduodenal administration might result in more targeted engraftment, depending on the gastrointestinal site being target. A pilot trial by Kang et al. (2017) on patients with psychiatric and GI symptoms found that colonoscopy‐based FMT led to improved microbiota diversity and symptom reduction.

Timing and frequency are also crucial. The IBD trial by Paramsothy et al. (2017) investigated repeated FMT using pooled donors. Although repeated FMT has been investigated in IBD, its optimal frequency and durability in AN remain unknown. Differences in nutritional status, intestinal physiology, and microbial composition may limit the applicability of these findings to patients with AN (Paramsothy et al. 2017).

Some protocols include antibiotics before FMT to reduce existing microbes population and improve donor strain colonization. However, this approach is controversial due to the risk of antibiotic resistance. A review in Clinical Infectious Diseases noted that while antibiotics such as vancomycin or rifaximin can temporarily reduce competing flora, but their long‐term safety in vulnerable populations such as patients with AN remains uncertain (Ademe 2020; Wang et al. 2019).

Lastly, recent evidence suggests that baseline microbial signatures may predict treatment response. For example, higher initial levels of certain bacteria, such as *Bifidobacterium* or *Akkermansia muciniphila*, have been associated with better metabolic outcomes following FMT in some populations. This supports the idea of using personalized microbiome profiling to guide donor selection or even develop custom microbial cocktails (Ding et al. 2022; Mocanu et al. 2021; Zhang et al. 2024). These findings highlight the complexity of optimizing FMT protocols for AN. Personalized approaches, including tailored donor selection, repeated dosing, and microbiota‐based stratification, may enhance treatment effectiveness, although their benefits in AN remain unconfirmed. While preliminary results suggest potential benefits, particularly in case reports and small pilot studies, larger controlled trials are needed to fully define the therapeutic role of FMT in anorexia nervosa.

### 1.13 Safety of Fecal Microbiota Transplantation in Anorexia Nervosa

Research into the safety of FMT as a therapeutic option for AN remains in its early stages. Although FMT has shown promise in restoring gut microbial diversity in other clinical contexts, its use in AN presents unique challenges due to the fragile physiological and immunological states of these patients.

Patients with AN often experience malnutrition‐related immune suppression, electrolyte imbalances, and gastrointestinal dysmotility, which may increase the risk of adverse events following FMT (Allegretti et al. 2016; Prochazkova et al. 2019). One of the earliest proposed studies in this area was developed at the University of North Carolina at Chapel Hill (UNC‐CH). It proposed administering FMT via nasogastric tube to patients with severe and enduring anorexia nervosa (SE‐AN) to assess safety and tolerability. However, this trial was later withdrawn before enrollment, and no results were published (ClinicalTrials.gov NCT03387041), illustrating the challenges of conducting interventional studies in this population. Patients with AN may be particularly vulnerable to potential complications from FMT due to severe undernutrition, impaired immune defenses, altered gut barrier function, electrolyte instability, and underlying medical fragility. Although serious adverse events have not been consistently reported in the limited case literature, these observations are insufficient to establish the safety of FMT in AN. The reason for UNC trial's withdrawal should not be assumed without documented evidence. Individuals with extremely low BMI, chronic starvation, bradycardia, micronutrient deficiencies, or intestinal permeability abnormalities may face an increased risk of infectious complications, microbial translocation, electrolyte shifts, or unpredictable metabolic responses following FMT. Therefore, rigorous pre‐screening, medical stabilization prior to FMT, and careful safety oversight should be required in future trials involving patients with AN. Importantly, much of the dysbiosis observed in AN may reflect starvation‐related adaptive microbial shifts rather than a disease‐specific pathogenic signature, further reinforcing the need for caution when attempting broad ecosystem manipulation through FMT. In addition to physiological concerns, potential psychological risks should also be acknowledged. Microbiota–brain interactions are complex and nonlinear, and large‐scale microbial shifts may not universally produce beneficial psychological effects. In individuals with high anxiety, rigidity, or obsessive–compulsive traits, which are common in AN sudden or broad microbial perturbation could theoretically worsen distress, somatic preoccupation, or anxiety symptoms.

This reinforces the need for careful psychological screening, appropriate treatment protocols, and close multidisciplinary monitoring when considering microbiome‐based interventions in this vulnerable population (Bastiaanssen and Cryan 2021). In a case report by Prochazkova et al. (2019), FMT was administered to a 37‐year‐old woman with severe and enduring AN. Following FMT, increased gut microbial diversity, enhanced intestinal barrier function, and increased levels of short‐chain fatty acids were observed. However, no significant clinical improvement in mood or eating behavior was reported. No serious adverse events related to FMT were reported in this single case. Given the single‐patient design and limited follow‐up, no conclusions regarding the safety or clinical efficacy of FMT in AN can be drawn (Prochazkova et al. 2019).

Animal studies have provided further insight into the potential effects of FMT. In activity‐based anorexia models, FMT from healthy mice improved weight gain and reduced anxiety‐like behaviors, suggesting that microbiota modulation could influence central nervous system function (Hata et al. 2019; Wilson et al. 2021). However, concerns persist about infection transmission, immune activation, and disruptions of host–microbiota equilibrium on especially in immunocompromised hosts. A meta‐analysis by Chinna Meyyappan et al. (2020) on FMT for psychiatric symptoms found transient improvements in anxiety and depression, although the findings were not consistently reproducible. Similarly, Afroz and Manchia (2023) emphasized the complexity of gut–brain interactions and the need for cautious implementation of microbiome‐targeted therapies in psychiatric populations. Importantly, findings from animal models and patients with other psychiatric disorders cannot be directly generalized to AN, particularly in individuals experiencing prolonged starvation and severe physiological instability. Although preliminary findings warrant further investigation, the safety and clinical efficacy of FMT in AN remain uncertain, particularly given the limited human evidence and lack of long‐term follow‐up. Until more data are available from adequately powered randomized controlled trials, FMT in AN should be conducted within closely monitored research settings.

### 1.14 Future of Fecal Microbiota Transplantation in Anorexia Nervosa

Rather than cross‐sectional comparisons, well‐designed longitudinal and interventional studies will be essential to disentangle causality and temporal directionality.

#### 1.14.1 Current Limitations in AN Treatment

AN remains one of the most challenging psychiatric disorders to treat effectively. Existing therapies include various forms of psychotherapy such as cognitive‐behavioral therapy and family‐based therapy, as well as nutritional rehabilitation. However, treatment outcomes vary, and relapse remains a significant challenge (Treasure 2018). Pharmacological treatments, particularly antidepressants, have also been used as adjunctive therapies but have shown mixed results (Kaye et al. 2004). Moreover, potential side effects and long‐term risks associated with antidepressants may limit their use in some patients with AN (Field et al. 2012). This treatment gap highlights the need to investigate for novel therapeutic strategies that address underlying pathophysiological mechanisms beyond psychological symptoms.

#### 1.14.2 The Role of Gut Microbiota and the Microbiota–Gut–Brain Axis

Emerging evidence suggests that alternation in the composition and function of intestinal microbial communities may contribute to the pathogenesis and persistence of anorexia nervosa (Lam et al. 2017). The gut microbiota interacts bidirectionally with the central nervous system through the microbiota–gut–brain (MGB) axis, which influences neurodevelopment, mood regulation, appetite control, immune modulation, and metabolic homeostasis (Cryan et al. 2019). Alterations in microbial composition may contribute to symptoms such as anxiety, depression, and gastrointestinal dysfunction, which are common in AN and may contribute to its chronicity. Thus, modulating the gut microbiota is being investigated as a potential therapeutic approach.

#### 1.14.3 Rationale for Fecal Microbiota Transplantation in AN

FMT involves the transfer of gut microbiota from a healthy donor into the gastrointestinal tract of a recipient, aiming to restore microbial diversity and function. Given the link between dysbiosis and AN symptomatology, FMT offers a theoretical and potentially transformative approach by directly modulating the intestinal flora. Initial case reports and small pilot studies have suggested improvements in gastrointestinal symptoms, nutritional status, and even psychological well‐being following FMT in patients with AN, supporting its potential as a therapeutic intervention (Prochazkova et al. 2021; Prochazkova et al. 2019; Roubalová et al. 2024).

### 1.15 Key Areas for Future Research and Development

#### 1.15.1 High‐Quality, Large‐Scale Clinical Trials

To establish FMT as a clinically validated treatment for anorexia nervosa, rigorous double‐blind, randomized controlled trials (RCTs) are essential. These trials would clarify FMT's efficacy, optimal patient selection criteria, dosing protocols, and safety profile. Given the complexity of AN and its heterogeneity, stratified clinical trials that consider disease stage, comorbidities, and microbiota profiles are needed to generate robust evidence that can guide clinicians (Lam et al. 2017; Prochazkova et al. 2021).

#### 1.15.2 Long‐Term Follow‐Up and Safety Monitoring

AN is a chronic disorder with a high risk of relapse. Therefore, long‐term follow‐up of patients receiving FMT is critical. Clinical trials and studies should monitor the stability of transplanted microbiota, durability of clinical improvements, and any late‐emerging adverse effects (Smillie et al. 2018). Continuous microbiome profiling and clinical assessments over months to years after FMT will provide insights into the longevity and potential risks, such as immune reactions or unintended microbial overgrowth (Kang et al. 2017).

#### 1.15.3 Optimizing Routes of Administration

Currently, FMT is delivered via various routes, including colonoscopy, nasogastric or nasojejunal tubes, capsules, or enemas. Each route presents different advantages and challenges regarding efficacy, patient tolerability, and safety. Comparative clinical trials focusing on AN patients could identify the most effective and patient‐friendly administration method, potentially improving adherence and clinical outcomes (Chinna Meyyappan et al. 2020).

#### 1.15.4 Determining Optimal Duration and Frequency

The appropriate duration and frequency of FMT treatments for AN remain unclear. Repeated or maintenance FMT sessions may be needed to sustain microbiota balance and symptom relief. Further research should aim to establish personalized treatment schedules based on symptom severity, microbiome response, and clinical endpoints to maximize benefits and minimize unnecessary interventions (Kang et al. 2017; Wilson et al. 2021).

#### 1.15.5 Precision Microbiota Profiling and Donor Selection

A major limitation is the variability in microbial taxa identified as altered in AN across studies, which complicates the choice of ideal donor microbiota. Advances in metagenomics, metabolomics, and bioinformatics could enable precise identification of dysbiotic microbial signatures in individual patients. This would allow for personalized donor selection or the development of synthetic microbial consortia tailored to restore specific deficits, while leveraging cutting‐edge techniques such as gene editing and microbial engineering to create highly stable and functional microbiotial supplements (Lam et al. 2017; Zmora et al. 2018).

#### 1.15.6 Synergistic Use With Other Microbial‐Targeted Therapies

Besides FMT, other approaches such as probiotics, prebiotics, symbiotics, and postbiotics also modulate gut microbiota and the MGB axis. Future studies could explore the combined use of these therapies with FMT to enhance efficacy, reduce treatment failures, and promote holistic gut ecosystem restoration. Understanding the interactions between these microbial‐targeted treatments may enable multimodal microbial therapies tailored for AN (Cryan et al. 2019).

Given the current early state of evidence, FMT should be conceptualized as a potential adjunctive therapy rather than a primary or standalone treatment. Integration of FMT within multidisciplinary AN care models is likely to be most appropriate—particularly alongside nutritional rehabilitation, psychotherapeutic approaches, and behavioral interventions that remain foundational to treatment. In clinical practice, GI dysmotility, abdominal discomfort, constipation, and impaired nutrient tolerance are significant barriers to refeeding in AN; therefore, future research could explore whether microbiota‐targeted interventions such as FMT could support these processes indirectly by improving gut function rather than attempting to treat core psychopathology directly (Tomba et al. 2021). Moreover, more accessible microbiome‐based approaches such as probiotics, prebiotics, synbiotics, or postbiotics may serve as more feasible near‐term options with lower risk and greater acceptability while the field continues to generate controlled evidence for FMT in AN (Smolinska et al. 2025).

Additionally, emerging evidence suggests that microbial diversity and functional capacity may remain partially blunted even after weight restoration and renourishment in AN, indicating that spontaneous microbiome recovery may not fully occur even once BMI is normalized (Monteleone et al. 2021). This raises the possibility that a more appropriate therapeutic window for FMT or other microbiota‐directed interventions may be during later stages of recovery after medical stabilization and nutritional rehabilitation—when the gut environment may be better equipped to support durable microbial engraftment, rather than during severe acute malnutrition.

FMT is unlikely to serve as a standalone therapy for anorexia nervosa. AN is a multifactorial disorder involving psychological, metabolic, and social dimensions that require comprehensive and multimodal treatment approaches. FMT could be investigated as an adjunctive modality alongside established psychotherapeutic, nutritional, and pharmacological interventions to improve the best patient outcomes. The future standard of care may involve personalized medicine combining gut microbiota modulation with traditional therapies, supported by advances in microbiome science (De Clercq et al. 2019; Prochazkova et al. 2021).

### 1.16 Limitations of Current Evidence and Interpretation

It is important to emphasize that current human evidence for FMT in AN consists almost entirely of case reports and very small uncontrolled pilot studies. Therefore, all interpretations remain preliminary and non‐causal. Most current human evidence supporting FMT in anorexia nervosa is derived from isolated case reports and very small, uncontrolled pilot studies. These observations are useful for hypothesis generation but cannot establish efficacy, causality, dose–response, or long‐term benefit. Reported improvements in weight, gastrointestinal function, or psychological state may reflect concurrent treatments (including nutritional rehabilitation), placebo influence, or natural fluctuations in illness course. Proposed mechanistic interpretation, such as SCFA restoration, immune modulation, or vagal pathway influence, remains speculative and are primarily extrapolated from animal models or indirect biomarkers rather than proven causal pathways in human AN. Therefore, the current state of research should be viewed as preliminary rather than conclusive. Future randomized, controlled, adequately powered human trials with standardized donor selection, dosing, engraftment metrics, and longitudinal clinical outcomes are required to draw reliable clinical conclusions. Due to the imitated availability of high‐quality randomized controlled trials or large prospective human cohorts in this field, some cited studies provide older and low‐tier evidence; therefore, their findings should be interpreted with appropriate caution. Furthermore, no formal risk‐of‐bias assessment was conducted, which limits our ability to systematically evaluate the methodological quality of the included studies.

Additionally, much of the mechanistic rationale currently depends on animal models, which do not fully recapitulate the physiological, psychological, and sociocultural complexity of AN in humans. Behavioral changes observed in rodents following microbiota transfers should not be assumed to represent equivalent therapeutic effects in people. These preclinical results should be interpreted as exploratory mechanistic evidence rather than predictive clinical evidence.

## 2 Conclusion

Dysregulation of the microbiota–gut–brain (MGB) axis may contribute to the pathophysiology of AN. Emerging evidence suggests that altered gut microbial composition may influence mood, appetite regulation, and stress responses through microbial metabolites, immune modulation, and vagal nerve signaling. SCFAs, tryptophan metabolism and altered gut permeability may contribute to the restrictive behaviors and psychological distress observed in AN. FMT is being investigate as a potential therapeutic strategy that may help restore microbial balance and influence signaling within the MGB axis.

Preliminary observations suggest FMT may be associated with improved microbial diversity and better GI tolerance in AN, alongside occasional reports of modest weight gain. However, evidence for psychiatric benefits remains limited and inconclusive. Future controlled clinical trials are required before therapeutic recommendations can be made or FMT can be implemented in routine clinical practice. The long‐term efficacy, durability, and safety of FMT in AN also remain largely unknown and required rigorously evaluation.

Personalized approaches, such as donor selection based on microbial profiles, optimization of treatment duration and combination of FMT with dietary, psychotherapeutic, or probiotic interventions, may warrant further investigation. FMT should be viewed not as a standalone treatment, but as a potential adjunctive therapy to existing multimodal care. To evaluate the therapeutic potential of FMT in AN, future clinical research should focus on elucidating the specific microbial taxa and functions most relevant to symptom modulation, identifying reliable biomarkers for treatment response, and conducting well‐powered randomized controlled trials. As our understanding of the MGB axis deepens, microbiota‐based interventions may offer additional opportunities for individualized treatment targeting the somatic and neuropsychiatric dimensions of AN.

## Author Contributions

**Shawn L. Kwok: **investigation (literature review), writing – original draft, writing – review & editing. **Yu‐Chen Xiao: **investigation (literature review), editing, and visualization (figure preparation). **Xian‐Bin Cai: **conceptualization, investigation (literature review), supervision, and funding acquisition. All authors reviewed and approved the final version of the manuscript for publication.

## Funding

This study is supported by 2023 Talent Program of the First Affiliated Hospital of Shantou University Medical College, Wu Jieping Medical Foundation Special Research Funding(funding number:2024).

## Ethics Statement

Ethical approval was not required for this review, as the study was based entirely on previously published literature and did not involve the direct participation of human subjects or animals.

## Conflicts of Interest

All authors declare no conflicts of interest.

## Declaration

All authors played a significant role in reviewing the literature and drafting the manuscript

## Data Availability

No new data were generated or analyzed for this review. All information included in this article was obtained from previously published studies cited in the manuscript.
